# Human immunodeficiency virus type 1 Vpr: oligomerization is an essential feature for its incorporation into virus particles

**DOI:** 10.1186/1743-422X-7-119

**Published:** 2010-06-07

**Authors:** Narasimhan J Venkatachari, Leah A Walker, Oznur Tastan, Thien Le, Timothy M Dempsey, Yaming Li, Naveena Yanamala, Alagarsamy Srinivasan, Judith Klein-Seetharaman, Ronald C Montelaro, Velpandi Ayyavoo

**Affiliations:** 1 Department of Infectious Diseases and Microbiology, Graduate School of Public Health, University of Pittsburgh, Pittsburgh, PA, USA; 2 School of Computer Science, Carnegie Mellon University, Pittsburgh, PA, USA; 3 Department of Structural Biology, University of Pittsburgh, Pittsburgh, PA, USA; 4 NanoBio Diagnostics, West Chester, PA, USA; 5 Vaccine Research Center, University of Pittsburgh, Pittsburgh, PA, USA

## Abstract

HIV-1 Vpr, a nonstructural viral protein associated with virus particles, has a positive role in the efficient transport of PIC into the nucleus of non-dividing target cells and enhances virus replication in primary T cells. Vpr is a 96 amino acid protein and the structure by NMR shows three helical domains. Vpr has been shown to exist as dimers and higher order oligomers. Considering the multifunctional nature of Vpr, the contribution of distinct helical domains to the dimer/oligomer structure of Vpr and the relevance of this feature to its functions are not clear. To address this, we have utilized molecular modeling approaches to identify putative models of oligomerization. The predicted interface residues were subjected to site-directed mutagenesis and evaluated their role in intermolecular interaction and virion incorporation. The interaction between Vpr molecules was monitored by Bimolecular Fluorescence complementation (BiFC) method. The results show that Vpr forms oligomers in live cells and residues in helical domains play critical roles in oligomerization. Interestingly, Vpr molecules defective in oligomerization also fail to incorporate into the virus particles. Based on the data, we suggest that oligomerization of Vpr is essential for virion incorporation property and may also have a role in the events associated with virus infection.

## Background

HIV-1 *vpr *gene encodes a protein of 96 amino acids with a predicted molecular weight of 14 kDa, which is conserved in both HIV and SIV [1]. Vpr is packaged into assembling virions by binding to the p6 domain of viral p55^Gag ^precursor protein. The presence of a functional Vpr is necessary for the efficient translocation of the pre-integration complex (PIC) into the nucleus and subsequent infection of primary monocytes/macrophages and other non-dividing cells [2-4]. Analysis of HIV-1 accessory genes (including *vpr*) in long-term non-progressors and asymptomatic patients suggests that defects in accessory genes are related to non-progressive status [5,6]. In this regard, the presence of defective or mutated *vpr *quasispecies has been shown to be associated with long-term non-progressive mothers [6-8]. Though *vpr *is selected against in tissue culture, selection for an intact Vpr occurs *in vivo *[9,10]. This finding suggests that *vpr *is required for optimal virus production and pathogenesis *in vivo *[11]. These observations clearly indicate the importance of Vpr in viral pathogenesis and disease progression.

HIV-1 Vpr is known to oligomerize both *in vitro *and *in vivo *[12,13]. This has been demonstrated by using cells in which Vpr was expressed either in the context of transfection of plasmid DNAs or through virus infection. Similar observations have also been reported with the purified Vpr protein generated using the prokaryotic expression system. Vpr has been shown to exist as dimers, trimers, tetramers and higher order multimers [13]. In general, protein oligomerization is thought to be an advantageous feature for the stability of the protein, interaction/binding with other proteins, allosteric control and the establishment of higher-order complexity [14]. HIV-1 Vpr, a non-structural protein, is incorporated into the virus particles and possesses several characteristic features that are known to play important roles in HIV-1 replication and disease progression. Vpr interacts with both viral and cellular host proteins, which are essential for Vpr-mediated functions. For instance, Vpr interacts with Gag-p6 and packages in the virus particles and virion-incorporated Vpr is known to positively regulate infection of non-dividing cells and enhance virus production in T cells [4,11,15,16]. However, it is not clear whether oligomerization of Vpr is required for virion incorporation and/or for its interactions with cellular proteins.

Vpr also has a well-defined role in apoptosis, cell cycle arrest and dysregulation of immune functions [17-19]. Many of the Vpr functions are carried out by virion-associated Vpr similar to *de novo *synthesized Vpr, suggesting that incorporation of Vpr into virus particles is an important event in HIV-1 biology. While the structure of Vpr based on X-ray crystallography is not yet available, biochemical analysis and NMR studies suggest that Vpr is composed of three alpha helices connected by loops [13,20-22]. Site-directed mutagenesis studies targeting single residues in Vpr indicated that amino acids in the N terminal region including the helical domains are essential for stability and virion incorporation and a region comprising the Helix III and the C terminal region determines the nuclear transport of Vpr [23-26]. With respect to oligomerization, it has been suggested that a leucine-zipper type mechanism is likely involving helix III based on the analysis of a peptide corresponding to the C-terminal region by NMR [27]. However, the structure of helix III in the peptide is different from that observed in the full-length protein [20]. Furthermore, mutagenesis studies have implicated additional amino acids in the hydrophobic core of the protein [12] in addition to a direct role for residue 44 in oligomerization through deletion. Thus, the roles of specific domains and residues involved in oligomerization are not yet defined.

To gain a better understanding of Vpr oligomerization and its role in virion association, we have utilized Bimolecular Fluorescence complementation (BiFC) analysis. This involves a chimeric protein strategy in which HIV-1 Vpr is fused to either N- or C-terminus fragment of the Venus protein. Upon expression and formation of dimers in live cells the Venus will emit fluorescence that can be detected by microscopy and flow cytometry. Such an approach combined with specific alterations in Vpr based on NMR structure and modeling have allowed us to evaluate the domains and residues essential for Vpr oligomerization and its relevance in virion incorporation. The results from the studies presented here indicate that Vpr molecules with distinct mutations in helical domains I, II and III dysregulate Vpr oligomerization and alter the ability of Vpr to incorporate into virus particles.

## Materials and Methods

### Cells and plasmid

HeLa, and 293T cells were grown in DMEM supplemented with 10% FCS, 1% glutamine and 1% penicillin-streptomycin. HeLa cells were obtained from NIH, AIDS reagent program. Proviral construct pNL43ΔVpr was generated by mutating the start codon of Vpr and verified by sequencing and westernblot analysis. Vpr expression plasmids were generated as described before [28]. All the mutant constructs were sequenced to verify the integrity of the mutations. For BiFC assays, sequences encoding the amino (residues 1 to 173, VN) or carboxyl (residues 155 to 238, VC) fragments of Venus fluorescence protein (template generously provided by Dr. Ronald Montelero, University of Pittsburgh) were fused to the N terminus of HIV-1 Vpr via a six-alanine linker and HA-tag for detection. All plasmids were isolated using a QIAGEN Maxiprep kit (QIAGEN, Valencia, CA), and the specific mutations were confirmed by DNA sequencing.

### Vpr dimer interface using docking model

Structural dimer models of Vpr were generated using ClusPro [29,30] and RosettaDock software [31] based on the full length monomer NMR structure of Vpr (PDB id:1m8l) [20]. First an approximate orientation of the dimer was obtained using the ClusPro server. The homo-multimeric docking option and DOT method [32,33] starting with 25000 initial conformations were employed. Of the resulting 10 best docking conformations, two models comprising antiparallel and parallel conformation based on best fit to the experimental data were selected. Each of these conformations was used as input into the Rosetta Dock server as the starting conformations for refinement via local docking of [31]. The hexamer model was generated using Cluspro server based on the monomer structure that comprised residues 15-78. The flexible N and C-termini were excluded since their inclusion resulted in unfeasible structural models where N and C termini formed the interface. All models are available upon request.

### Expression of Vpr molecules by immunoblot

HEK293T cells were cotransfected with Vpr expression plasmid using L Lipofectamine. Forty-eight hours post-transfection, Cells were washed twice with PBS and lysed in RIPA buffer containing 50 mM Tris (pH 7.5), 150 mM NaCl, 1% Triton X-100, 1 mM sodium orthovanadate, 10 mM sodium fluoride, 1.0 mM phenylmethylsulfonyl fluoride, 0.05% deoxycholate, 10% sodium dodecyl sulfate, aprotinin (0.07 trypsin inhibitor unit/ml), and the protease inhibitors leupeptin, chymostatin, and pepstatin (1 μg/ml; Sigma). Cell lysates were clarified by centrifugation, and total cell lysates (50 μg) were separated on a 12% sodium dodecyl sulfate-polyacrylamide gel (SDS-PAGE) electrophoresis gel, transferred, and immunoblotted with anti-HIV-1 p24 for Gag and anti-HA for Vpr. The blots were developed using an ECL kit (Amersham Biosciences, Piscataway, NJ).

### BiFC Flow cytometry

Thirty-six hours post transfection 293T cells were washed with PBS and fixed in 3.7% formaldehyde at room temperature for 15 minutes, and washed and resuspended in 200 μl of FACS buffer. Samples were analyzed using Epics-XL (Beckman Coulter, Miami, FL) with minimum of 40000 gated events acquired for each sample, and percent BiFC positive cells was calculated using Flow Jo software.

### Immunofluorescence

HeLa cells were used to perform all immunofluorescence and microscopy assays. Thirty-six hours post transfection, cells were washed with PBS and fixed in 3.7% formaldehyde at room temperature for 10 minutes, and washed and permeabilized with 0.5% Triton X-100 for an additional 10 minutes. After washing 3 times with PBS, cells were blocked with 5% BSA at room temperature for 1 hour followed by incubation with primary antibody (HA or Vpr; 1:200 dilution) for 1 hour at room temperature and incubated with rabbit anti-mouse or mouse anti-rabbit IgG Rhodamine (RRX) (1:400; Jackson ImmunoResearch, West Grove, PA) for 1 hour at room temperature. Cells were mounted with VECTASHIELD mounting media containing DAPI (Vector Laboratories, Burlingame, CA). Immunofluorescence analysis was performed using a fluorescence microscope with Nikon SPOT camera (Fryer, Huntley, IL) and images were processed using MetaMorph software (Universal Imaging Corporation, Downington, PA).

### Vpr-Gag interaction by BiFC and virion incorporation

Oligomerization of Vpr in live cells was evaluated by BiFC methods. HeLa cells were seeded in 6-well plates with glass slides and transfected with combinations of Vpr^wt^, Vpr mutants and Gag expression plasmids with their respective VN or VC combinations or with control plasmids using Lipofectamine. Forty hours post-transfection, cells were washed with PBS and fixed with 2% paraformaldehyde. Cells were mounted with VECTASHIELD containing DAPI (Vector Laboratories, CA) and fluorescence was detected using Nikon inverted fluorescence microscope with appropriate filters. The remaining cells in the plates were analyzed by flow cytometry followed by cell quest software to measure the percent fluorescent positive cells. Ability to Vpr mutants to interact with Gag and package into virus particles was assessed by monitoring the presence of Vpr in virus particles. Briefly, cells were cotransfected with pGag and pVpr mutants or vector plasmid as described before. Forty hours post transfection, supernatant was collected, spun at 2000 rpm to remove cell debris and passed through 0.22 μM filter to remove aggregates and cell debris. Filtered supernatant was ultracentrifuged to pellet the virus particles and used in SDS-PAGE followed by immunoblot with anti-Gag (p24) and anti-Vpr antibody. Similarly, cells were pelleted and cell lysates were used to detect the presence and expression of Gag and Vpr in transfected culture.

## Results

### Construction and characterization of chimeric Vpr for oligomerization studies using BiFC analysis

**Figure 1:**
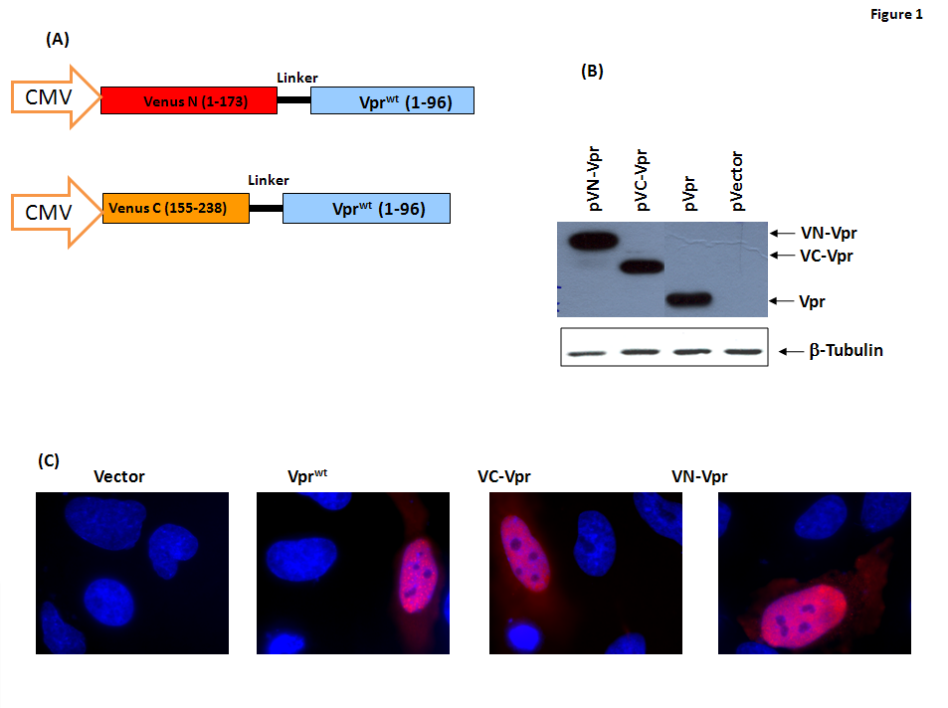
**Construction and characterization of Vpr plasmids for oligomerization studies in live cells using BiFC analysis**. **(A) **Schematic representation of Vpr^wt ^fused with Venus-N terminal or Venus-C-terminal fragments. **(B) **Expression of Venus-C Vpr^wt ^and Venus-N Vpr^wt ^was assessed in HEK293T cells by transient transfection. HEK293T cells were transfected with Venus-N-Vpr^wt ^or Venus C-Vpr^wt ^expression plasmids or Vector control plasmid, and assessed by Western blot. **(C) **Subcellular localization pattern of Vpr^wt ^or Venus-Vpr^wt ^fusion proteins was assessed in HeLa cells by transient transfection. HeLa cells were transfected with Vpr^wt ^or Venus C - Vpr^wt ^or Venus N - Vpr^wt ^expression plasmids or Vector control plasmid, and assessed by Immunofluorescence for subcellular localization pattern using Vpr-specific antibody. Figure represents one of five independent experiments (n = 5) with similar results.

The ability of Vpr to oligomerize was demonstrated by biochemical methods using bacterially produced or chemically synthesized Vpr protein and/or peptides [15]. A recent study has shown that Vpr forms dimers and oligomers in relevant eukaryotic cells by using fluorescence spectroscopy and imaging analysis [12]. These observations have prompted us to evaluate the requirement of specific domains/residues of Vpr in the oligomerization of the molecules. It is important to note that, although oligomerization in live cells has been reported previously, the assays used for this purpose were mostly qualitative in nature. We have selected Bimolecular Fluorescence Complementation (BiFC) system based on Venus as a reporter protein to quantify the interactions between Vpr monomers. Chimeric proteins containing Vpr and N- or C- terminus fragments of the Venus reporter are to result in reconstitution of a functional Venus protein with fluorescence. Since the assay does not distinguish between dimers and oligomers containing more than two Vpr proteins, we will use the term oligomerization to describe the contact between at least two Vpr proteins throughout the manuscript. Briefly, Vpr from NL43 (which will be referred as Vpr^wt^) was cloned downstream of its N terminus (1-173 aa) or C terminus (155-238) of Venus fluorescent protein as described [34]. The schematic representation of the constructs is presented in Fig. 1A. The recombinant plasmids were assessed for expression of the correctly sized protein products by transient transfection in HEK293T cells followed by immunoblot assay. The results shown in Fig. 1B indicate that a chimeric (Venus-Vpr) protein was expressed in cells transfected with the respective plasmids. The chimeric protein of expected size was detected by antibody against Vpr. The steady state expression levels of chimeric proteins were similar to that of wild type Vpr. Next, we also analyzed the subcellular localization pattern of the chimeric proteins in comparison to the untagged wild type Vpr by using Vpr specific antibody. Such an analysis showed that fusion of N- and C-terminal fragment of Venus reporter did not alter Vpr localization (Fig. 1C). Together these results indicate that fusion of chimeric molecules (VC and VN) did not alter the expression or subcellular distribution of Vpr.

**Figure 2:**
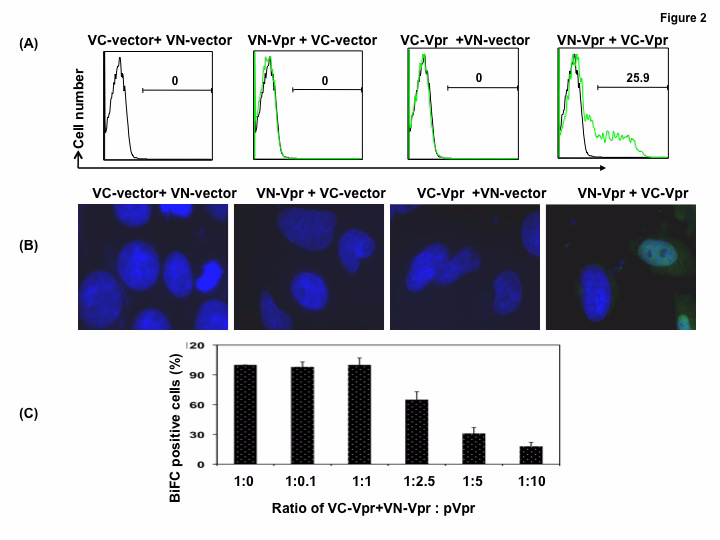
**Visualization of Vpr oligomerization by (A) Flow cytometry or (B) Fluorescent microscopy**. **(A) **Quantitative analysis by flow cytometry of Venus fragment complementation in HeLa cells transfected with VC-Vpr and VN-Vpr or with control plasmid. Thirty-six hours posttransfection, cells were harvested and analyzed by flow cytometry to determine the percentage of cells positive for BiFC fluorescence. Results represent the means of five independent experiments. **(B) **Subcellular localization of the BiFC complex: HeLa cells grown on glass coverslips were cotransfected with VC and VN plasmids, VN-Vpr^wt ^and VC-Vpr^wt ^or VN- Vpr^wt ^or VC- Vpr^wt ^with control plasmid pairs using Lipofectamine. At 36 hours post-transfection, cells were fixed, stained with DAPI and imaged at 60× magnification. **(C) **To confirm the specificity of VC and VN based oligomerization, VC-Vpr (0.5 ug) and VN-Vpr (0.5 ug) plasmids were cotransfected with increasing concentrations of empty vector or Vpr expression plasmid. Thirty-six hours post transfection cells were fixed and assessed by flow cytometry. BiFC positive cells (%) were calculated in each treatment compared with the control. Figure represents one of five independent experiments (n = 5) with similar results.

To monitor oligomerization in live cells, HeLa cells were cotransfected with equal amounts of Venus-C Vpr and Venus-N Vpr plasmids or each plasmid with backbone Venus C or N-terminal fragment containing vector as a control. Vpr oligomerization was monitored thirty-six hours posttransfection by flow cytometry (Fig. 2A) and by fluorescence microscopy (Fig. 2B). Results indicate that cells transfected with both VC-Vpr and VN-Vpr exhibit a positive signal (26% of BiFC positive cells) that is detected by both approaches, whereas VC and VN vector, VC-Vpr or VN-Vpr with a vector control plasmid did not show any signal. Similar results were observed in Jurkat cells transfected with Vpr BiFC plasmids (data not shown). To ascertain the specificity of Vpr-Vpr interactions, we have also carried out a competition experiment in which vector backbone or untagged Vpr is expressed along with chimeric VC-Vpr and VN-Vpr. As expected, the inclusion of untagged Vpr has resulted in a diminished BiFC signal in a dose dependent manner (Fig. 2C). Considering the BiFC positive cells in wells transfected with VC-Vpr and VN-Vpr plasmids against the vector backbone as 100%, we assessed the BiFC positive cells in a pool of cells transfected with a combination of three plasmids including pVpr. The addition of pVpr at a ratio of 1:1 (2.5:2.5 μg of DNA) to cells cotransfected with VC-Vpr and VN-Vpr did not reduce BiFC positive cells, whereas at a ratio of 1:2.5 there is a 40% reduction and it was reduced to less than 20% BiFC positive cells at 1:10 ratio. As increasing amount of untagged Vpr plasmid competed with VC and VN chimeras, percent positive BiFC cells was reduced. Cell viability was monitored in these cultures during the experimental period and no significant cell death was observed suggesting that the observed reduction in BiFC signal is due to the competition of untagged Vpr and not due to Vpr-induced apoptosis (data not shown). Together these results indicate that oligomerization of Vpr is specific and this technique would allow us to study the interaction in live cells more efficiently, especially in HIV-1 target cells.

### Structure-based prediction of the Vpr dimer and oligomeric interfaces

**Figure 3:**
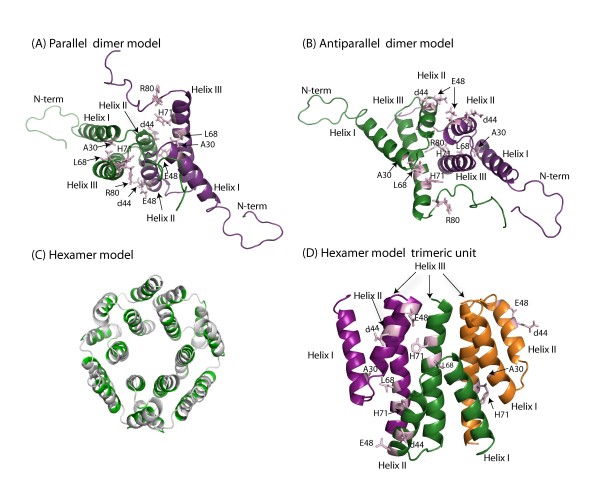
**Docked homo-oligomer models of Vpr**. **(A) **Parallel dimer model; **(B) **Antiparallel dimer model; (**C) **Hexamer model top view, green indicates hydrophobic residues; **(D) **Hexamer model side view, only the trimeric unit is shown. Residues that are selected for this study are indicated with arrows.

We employed computational approaches to derive hypotheses regarding putative *in vivo *dimer interfaces. Using the available full-length Vpr NMR protein structure (pdbid:1M8L), we built docking models using Cluspro [29,35] and Rosetta softwares [31]. The highly ranked models included both antiparallel and parallel orientations. In the majority of models, the interface comprised mainly residues from helices II and III. Fig. 3A and 3B show models with parallel and antiparallel orientations, respectively. Helix I faces away from the dimer interface in the models shown in Fig. 3. This leaves the possibility for helix I to mediate higher order oligomerization. Such a role is feasible because helix I has high coiled coil propensity (data not shown). The list of the interface residues in the dimer models together with the secondary structure is listed in Table 1. A residue is considered in the interface, if it has at least one atom within 5.0 Å distance to any atom. With the exception of R80 many of the selected residues are within the dimer interface. Next, to verify if higher order oligomerization states are compatible with the Vpr structure, we formed higher order models. A particularly plausible hexamer model is shown in Fig. 3C and Fig. 3D. This model was constructed from the monomer NMR structure by excluding the flexible N and C-termini. The predicted conformation included both parallel and antiparallel dimeric units. Hydrophobic residues were buried in the interfaces. In addition to helices II and III, helix I was part of the hexamer interfaces.

**Table 1:** HIV-1 Vpr residues involved in dimerization based on dimer models

Parallel dimer model	
**Secondary structure unit**	**Residues in the interface**
Helix 1	30-32,34
Loop 1	35, 36
Helix 2	37-42,44-46,48,49
Helix 3	56,59,60,63,64,67,68,70,71
C-terminus	84,85,91
	
**Antiparallel dimer model**	
**Secondary structure unit**	**Residues in the interface**
N-terminus	1,14,15
Helix1	18-19,22,34
Loop 1	35,36
Helix 2	38,41,42,44-46,48-50
Loop2	53
Helix 3	54-59,62-64,66,67,69-71,74
C-terminus	80,82-86,90-94

A residue is considered in the interface, if it has at least one atom within 5.0 Ǻ distance to any atom in the other chain.

### Role of predicted interface residues in Vpr oligomerization

**Figure 4:**
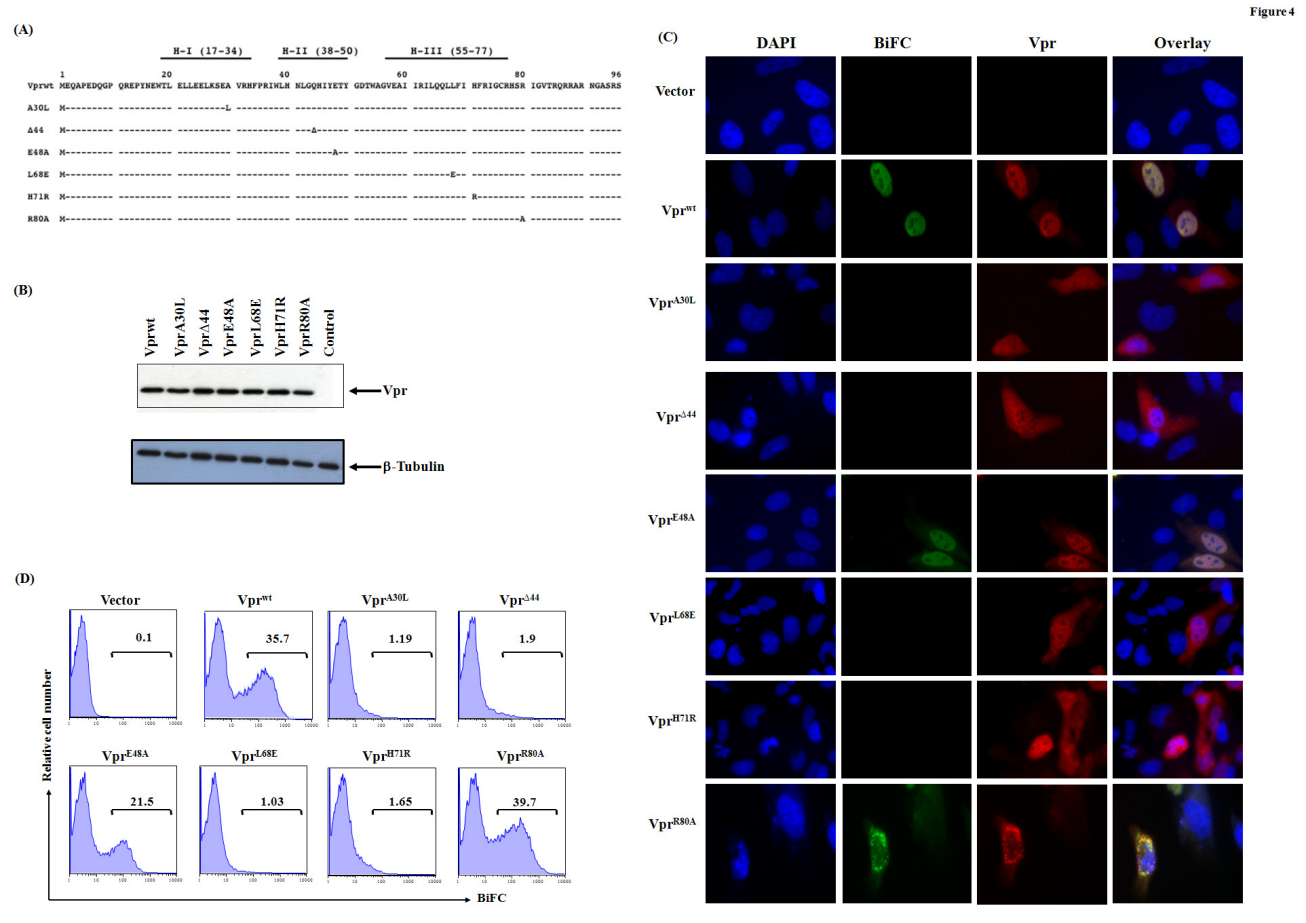
**Identification of residues required for Vpr oligomerization**. **(A) **Schematic figure depicting Vpr mutants selected for analysis. Substitution residue(s) are marked at appropriate place and a Δ represent deletion of a residue. NL43 sequence was used as wild type clone. **(B) **Expression of Vpr mutants was assessed in HEK293T cells by transient transfection. HEK293T cells were transfected with Vpr mutant expression plasmids or vector control plasmid and assessed for expression by western blot using antibodies against HA epitope to detect fusion protein. Tubulin was used as a loading control. **(C) **Visualization of oligomerization and subcellular distribution of Vpr mutant molecules was assessed by BiFC. HeLa cells were transfected with VC and VN combinations of Vpr mutants as described. Thirty-six hours posttransfection cells were stained for Vpr (shown in Red) and DAPI (blue) and imaged at 60× magnification. Figure represents one of five independent experiments (n = 5) with similar results.

Given that all three helices are predicted to be involved in oligomerization, we selected from the putative interface candidate residues for further biological evaluation. The residues targeted for mutational analysis are shown in the context of the NL43 sequence along with the helical domains in Fig. 4A. We have also included, Δ44 mutant as a positive control as this is already known to be defective in dimer formation [12] in addition to R80, which resides outside of the helical domains. Vpr mutant molecules were cloned in venus-C and venus-N construct and verified for expression in HEK293T cells by transfection followed by immunoblot analysis as shown for VN-Vpr constructs in Fig. 4B. Similar results were observed for VC-Vpr constructs (data not shown). Cell lysates were normalized for transfection efficiency and loaded equally. Results indicate that both VN and VC Vpr mutant chimeric molecules express the appropriate size protein. The expression level of all Vpr mutant molecules is comparable to wild type, with the exception of L68E (slightly lower with similar amount of DNA used); however level of VprL68E expression was equalized by increasing the amount of VprL68E plasmid used for transfection.

Next, we assessed the ability of Vpr mutants to form oligomers by BiFC analysis. The expression of Vpr molecules was also assessed from the same batch of cells by indirect immunofluorescence using HA antibody (to detect HA tagged Vpr) and compared with BiFC by microscopy (Fig. 4C). Results indicate that Vpr^wt^, E48A and R80A exhibited 35, 22 and 39% BiFC positive cells respectively, whereas mutants A30L, Δ44, L68E and H71R did not show BiFC positive cells, suggesting that these mutants are defective in oligomerization. The differences in percent BiFC in Vpr^wt^, E48A and R80A is due to difference in transfection efficiency and did not show statistical significance in multiple experiments. Importantly, staining for Vpr (panel Vpr in Fig. 4C) further confirmed the expression of Vpr protein suggesting that lack of dimerization is not due to lack of Vpr expression. We also assessed whether subcellular distribution of Vpr mutant molecules has any role in oligomerization. Both Vpr^wt^, and E48A showed uniform nuclear localization. The Vpr mutant R80A exhibited nuclear membrane distribution of venus fluorescence. On the other hand, BiFC negative mutants, A30L, Δ44, L68E and H71R showed both nuclear and cytoplasmic distribution suggesting that subcellular distribution of Vpr is different in oligomerization defective mutants in comparison to Vpr^wt^. Similar results were reported by other groups indicating that nuclear localization of Vpr is not absolutely necessary for Vpr mediated functions such as cell cycle arrest and virion incorporation [26,36].

### Relevance of oligomerization in Vpr-Gag interaction and virion incorporation of Vpr

**Figure 5:**
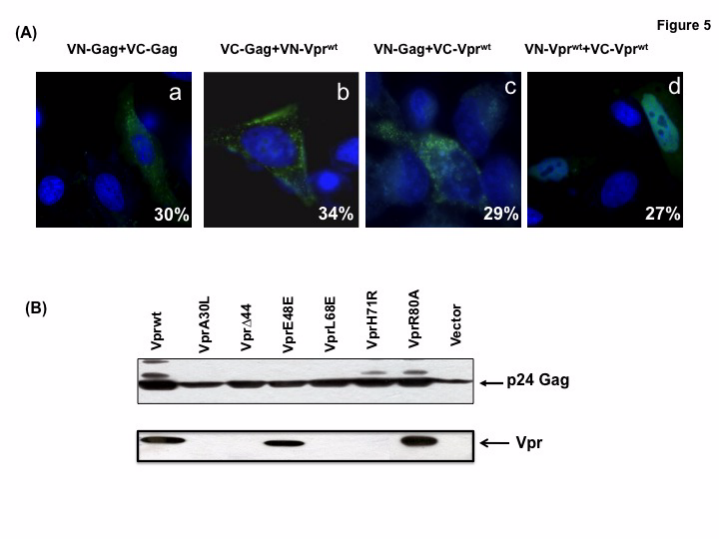
**Role of Vpr oligomerization in incorporation of Vpr into virus particles**. **(A) **Visualization of Vpr^wt ^and Gag interaction in HeLa cells by BiFC. HeLa cells were transfected with VN-Vpr^wt ^and VC-Gag expression plasmids. Thirty-six hours post transfection, cells were fixed, stained with DAPI and analyzed for presence and pattern of BiFC signal at 60× magnification. **(B) **Incorporation of Vpr mutant molecules into virus particles. Vpr plasmids were cotransfected with pNL43ΔVpr proviral plasmid in HEK293T cells. Forty-eight hours post-transfection, supernatant was collected, filtered, lysed and subjected to SDS-PAGE electrophoresis and evaluated for the presence viral proteins p24-Gag and Vpr using p24-specific and Vpr specific antibodies by western blot. Figure represents one of five independent experiments (n = 5) with similar results.

It is known that Vpr interacts with HIV-1 Gag specifically through the p6 domain and packages into the virus particles in significant quantities [15,37-40]. Therefore, we were interested in assessing whether Vpr-Gag interaction is detectable in BiFC based live cell assay using Venus-Gag and Venus-Vpr plasmids. Combination of venus-C and venus-N plasmids expressing either Gag or Vpr was cotransfected and evaluated for BiFC signal by flow cytometry and fluorescence microscopy (Fig. 5A). Flow cytometry analysis revealed specific oligomerization of Gag (30%) with distinct Gag distribution at the cell membrane (marked in Fig. 5A panel). Combination of venus-N-Gag and venus-C-Vpr^wt ^or venus-C-Gag and venus-N-Vpr^wt ^resulted in 34% and 29% fluorescent positive cells, respectively. There was no signal detected with appropriate control plasmid transfection suggesting that Vpr-Gag interaction is specific as reported previously [26,40]. Subcellular distribution of Gag-Gag interaction and Gag-Vpr interaction resulted in cytosolic and cytoplasmic membrane localization (Fig. 5A; panel a-c), whereas Vpr-Vpr interaction resulted in nuclear localization (Fig. 5A; panel d). Together, the fluorescence microscopic analyses reveal that Vpr interacts with Gag specifically at the cytoplasmic membrane as well as in the cytoplasm and this interaction results in differential localization of Vpr corresponding to virion incorporation and virus assembly.

Next, we assessed the ability of Vpr mutants to incorporate into virus particles. HEK293T cells were cotransfected with pNL43Δvpr proviral plasmid and Vpr expression plasmids or vector control. Vpr amount in cell lysate was quantitated by loading protein normalized for transfection efficiency and amount of Vpr in virus particles was quantitated by loading viral lysates normalized using Gag-p24 antigen, respectively (Fig. 5B). Presence of Vpr in virus particles was noted only in Vpr^wt^, E48A and R80A, whereas, A30L, Δ44, L68E and H71R did not show virion associated Vpr. However, all mutants showed equal amount of p24-Gag in the same sample. These results suggest that mutants A30L, Δ44, L68E and H71R are defective in virion incorporation and they did not alter/interfere with the virus production in the cells contransfected with NL43Δvpr proviral DNA and Vpr encoding plasmid. To further confirm that the lack of Vpr in virus particles is not due to lack of Vpr expression in these cells, cell lysates from the same samples were assessed for Vpr and p24 levels (data not shown). The results indicate that comparable levels of Vpr and p24 were observed in all cell lysates (Fig. 4B). Together theses studies indicate that Vpr oligomerization is an essential feature for virion incorporation.

To further confirm that Vpr mutants that are defective in virion incorporation lack the ability to interact with Gag, Vpr mutants were cotransfected with VN-Gag or VC-Gag with appropriate controls and assessed for BiFC (Table 2). Results indicate that VC/VN chimeric Vpr^wt^, E48A and R80A plasmids in combination with VC/VN-Gag showed measurable BiFC positive cells, whereas A30L, Δ44, L68E and H71R are negative for BiFC compared to positive control. Collectively, these studies indicate that Vpr mutants that are defective in virion incorporation are defective in Gag interaction and virion incorporation.

**Table 2:** Percent BiFC positive cells in wells transfected with a combination of Vpr and Gag expression plasmids.

Vpr plasmids	VC-Gag	VN-Gag
VC-Vpr^wt^	0	31
VN-Vpr^wt^	27	0
VC-VprA30L	0	0
VN-VprA30L	0	0
VC-VprΔ44	0	0
VN-VprΔ44	0	0
VC-VprE48A	0	29
VN-VprE48E	28	0
VC-VprL68E	0	0
VN-VprL68E	0	0
VC-VprH71R	0	0
VN-VprH71R	0	0
VC-VprR80A	0	33
VN-VprR80A	27	0

293T cells were cotransfected with combinations of VC and VN-chimeric Vpr and Gag expression plasmids as described in methods. Thirty-six hours post transfection cells were fixed and assessed by flow cytometry. BiFC positive (%) cells are presented in the respective combination. Results represent one of three independent experiments with similar results.

## Discussion

HIV-1 and 2 are members of lentivirus family of retroviruses and are grouped as complex retroviruses. The unique feature of this group in comparison to simple retroviruses is that the viral genome codes for several proteins in addition to the core structural proteins. In this regard, HIV-1 is known to code for six auxiliary proteins (Vif, Vpr, Tat, Rev, Vpu and Nef) besides the structural proteins. Previous studies have demonstrated that auxiliary proteins play an essential role in HIV-1 replication and pathogenesis [41]. Our laboratory has been interested for several years in evaluating the contribution of auxiliary proteins including Vpr. In this study, we have analyzed the requirement of sequences in Vpr essential for oligomerization feature of Vpr and its relevance to the functions of Vpr. Specifically, Vpr shares this feature with other auxiliary proteins such as Vif, Rev, Vpu, and Nef.

HIV-1 Vpr is a small oligomeric protein that plays an important role in HIV pathogenesis [5,17,19,23,42]. The underlying reasons for the selection of Vpr for the present studies are the following: (i) Vpr is a virion associated protein; (ii) Vpr plays a critical role for the replication of virus in macrophages and positively regulates viral replication in T cells; (iii) Vpr is a transcriptional activator of HIV-1 and heterologous cellular genes; (iv) Vpr inhibits proliferation of cells at G2/M phase; (v) Vpr induces apoptosis in diverse cell types including T cells and neurons; (vi) Vpr exhibits immune suppressive effects. Further, studies from non human primates and analysis of viral genes in long term non progressors suggest a correlation between defective Vpr and delayed progression of the disease [5,43,44]. More importantly, several Vpr-mediated functions are known to be induced by both cell-associated and virion-associated Vpr [17,18,45]. Together these studies point out the biological significance of virion associated factors and its role in early events associated with virus infection. Therefore, understanding the role of oligomerization in Vpr functions and disease progression may provide useful information for the development of therapeutics against HIV-1 targeting Vpr.

The oligomerization feature of Vpr was evaluated by using a complementation system based on Venus protein as a reporter. A strategy involving the generation of chimeric Venus-Vpr protein has allowed us to monitor oligomerization in live cells. This system has the sensitivity to detect Vpr-Vpr and Vpr-Gag interactions. Studies on the oligomerization of Vpr, analyzed by site-specific mutagenesis, identified some residues located in specific domains of Vpr. However, there is no information available regarding the dimer interface structure of Vpr. To address this, we have utilized the available NMR structure and modeling approaches to identify the residues that form putative dimer and oligomer interfaces of Vpr. Deletion of residue at position 44, which is predominantly glutamine (Q), is known to be oligomerization defective and was used as a control in these experiments. We reasoned that moderate replacements, in particular those mostly affecting size, should show significant biological effects if these residues form part of the interfaces. Thus, for A30 and L68 we chose L and E replacements, respectively, conserving hydrophobicity but changing the size of the position. On the other hand, at position H71, we chose R, maintaining the positive charge. Mutant Vpr molecules were generated containing alterations in the selected residues. The results regarding the expression and steady state level of Vpr indicated that mutants lacking the ability to oligomerize exhibit a pattern similar to that of wild type Vpr. This observation suggests that monomeric Vpr molecules are stable in cells, which is in agreement with an earlier report [36].

To interpret the mutagenesis data in structural terms, we created structural models of the Vpr dimer and oligomers through molecular docking based on the available full length monomer structure [20]. Our models are shown in Figure 3, highlighting the positions of the residues that resulted in oligomerization defective properties of Vpr *in vivo*. The docked models revealed propensities for both parallel and antiparallel orientations of the monomers within a dimer indicating that both of the conformations are plausible. Regardless of orientation, helices II and III constitute the dimer interface in the majority of the models. In the parallel orientation, residues 30, 44, 68, 71, all of which fail to oligomerize when mutated, even with the conservative substitutions chosen, were part of the interface; while in the antiparallel orientation residues 44, 68 and 71 contributed to the binding energy in the interface. Further supporting our predicted interfaces, recent studies of Vpr mutants I60A and I67A indicated that these residues play a major role in trafficking the Vpr to the nuclear rim [12]. Both of these residues are part of the interface in the predicted parallel conformation, whereas I60 is part of the antiparallel model. In agreement with the experimental results presented here suggest that both of these two dimer conformations are likely to occur *in vivo*. The parallel orientation fits the experimental data better, but further studies will be needed to fully differentiate between the two models. Furthermore, assays are needed that can clearly distinguish dimers from higher order oligomers. Based on the combined modeling and experimental results, we propose that dimerization primarily involves helices II and III, while oligomerization includes helix I also. The fact that the Vpr mutation A30L reported here and the recently studied nuclear rim localization defective mutant L23F [12] are located in helix I supports the proposed role of helix I in oligomerization. We predict that because helix I in the dimer models faces away from the dimer interface, it may play a pivotal role in mediating higher order Vpr-Vpr interactions. We therefore built models for higher order multimeric forms, and a hexamer model (Fig. 3C and Fig. 3D) exhibited an interface where all the three helices participated in interaction interfaces. In this hexamer model, both parallel and antiparallel dimeric units were present and the hydrophobic residues faced to interior of the helices (Fig. 3D).

HIV-1 Vpr is one of the non-structural proteins that is packaged in significant quantities in virus particles. Virion-associated Vpr is present in the infected cells prior to *de novo *synthesis and is known to cause the host cellular dysfunctions during early infection [17,19,42]. Studies have indicated that the p6 domain of Gag is critical for the incorporation of Vpr into virus particles [15,39,40,46]. However, it is not clear whether Vpr oligomerization is a prerequisite for virion incorporation. As expected, chimeric Venus containing wild type Vpr and chimeric Venus containing Gag resulted in the reconstitution of Venus with fluorescence suggesting an interaction between these two proteins. On the other hand, chimeric Venus containing mutant Vpr failed to interact with Gag. Vpr mutants that showed oligomerization negative phenotype also failed to incorporate into virus particles. Several studies have reported that virus particle contains between 14-275 molecules of Vpr in comparison to approximately 2500-2750 molecules of Gag protein depending on the system and methods used [38,47]. This suggests that low amount of Vpr to Gag may be due to the interaction restricted to the specific configuration of Gag. Several studies evaluated Vpr-Gag interaction and reported that helical domain I (residues E25 and A30), P35 and helical domain III (isoleucine-leucine residues) in Vpr are required for interaction with Gag, thus virion incorporation [26,39,48]. Our results on Vpr-Gag interaction using BiFC (Table 2) are in agreement with these studies and further supports the utility of BiFC assay for evaluating the interactions of Vpr with interacting partner proteins. Oligomerization defective mutants, A30L, Δ44, L68E and H71R lack the ability to incorporate into the virus particles, suggesting that Vpr oligomerization might be directly linked to virion associated Vpr functions, pathogenesis and disease progression. A very recent publication further confirms our findings that Vpr oligomerization is required for interaction with Gag and oligomerization deficient mutants of Gag interacted with Vpr [49].

An understanding of HIV-1 Vpr functions and its properties, in our view, is likely to shed light on the mechanisms involved in Vpr incorporation into the virus particle and how oligomerization feature influences infection of non dividing target cells. Although Vpr is not essential for virus replication in *in vitro *studies using established cell lines, it is well established that virion- associated Vpr play a major role in macrophage infection by aiding the transport of PIC into the nucleus [50,51]. More importantly, virion-associated Vpr is known to mediate several host cellular events and immune evasive functions that are very similar to *de novo *synthesized Vpr [18,52,53]. This further bolsters the significance of virion-associated proteins present both in infectious and noninfectious particles and their role in HIV-1 pathogenesis. These studies further support the idea of developing potential therapeutic agents including small molecules against Vpr-Vpr interaction, Vpr-Gag interaction, virion incorporation and virion associated Vpr induced host cell dysregulation to combat HIV-1 infection.

## Competing interests

The authors declare that they have no competing interests.

## Authors' contributions

NJV, OT, AS, JKS and VA conceived and designed the experiments, NJV, LAW, OT and YL performed the experiments, NJV, LAW, OT, AR, NY, AS, JKS, RCM and VA analyzed the data, NJV, LAW, OT, TL, TMD, AR and NY contributed reagents/materials/analysis tools, NJV, OT, AS, JKS and VA wrote the paper. All authors have read and approved the final manuscript.
